# Disinfection of Neonatal Resuscitation Equipment in Low-Resource Settings: The Importance, the Reality, and Considerations for the Future

**DOI:** 10.3390/ijerph18137065

**Published:** 2021-07-01

**Authors:** Anne M. White, Dominic Mutai, David Cheruiyot, Amy R. L. Rule, Joel E. Mortensen, Joshua K. Schaffzin, Beena D. Kamath-Rayne

**Affiliations:** 1 University of Minnesota Masonic Children’s Hospital, University of Minnesota, Minneapolis, MN 55454, USA; 2 Tenwek Hospital, Bomet 20400, Kenya; mutai.dominic3@gmail.com (D.M.); kiplaa2009@yahoo.com (D.C.); 3 Cincinnati Children’s Hospital Medical Center, Cincinnati, OH 45229, USA; amy.rule@cchmc.org (A.R.L.R.); joel.mortensen@cchmc.org (J.E.M.); joshua.schaffzin@cchmc.org (J.K.S.); 4 American Academy of Pediatrics, Itasca, IL 60143, USA; bkamathrayne@aap.org

**Keywords:** neonatal mortality, neonatal infection, reprocessing, disinfection, global child health

## Abstract

Preventable neonatal deaths due to prematurity, perinatal events, and infections are the leading causes of under-five mortality. The vast majority of these deaths are in resource-limited areas. Deaths due to infection have been associated with lack of access to clean water, overcrowded nurseries, and improper disinfection (reprocessing) of equipment, including vital resuscitation equipment. Reprocessing has recently come to heightened attention, with the COVID-19 pandemic bringing this issue to the forefront across all economic levels; however, it is particularly challenging in low-resource settings. In 2015, Eslami et al. published a letter to the editor in *Resuscitation*, highlighting concerns about the disinfection of equipment being used to resuscitate newborns in Kenya. To address the issue of improper disinfection, the global health nongovernment organization PATH gathered a group of experts and, due to lack of best-practice evidence, published guidelines with recommendations for reprocessing of neonatal resuscitation equipment in low-resource areas. The guidelines follow the gold-standard principle of high-level disinfection; however, there is ongoing concern that the complexity of the guideline would make feasibility and sustainability difficult in the settings for which it was designed. Observations from hospitals in Kenya and Malawi reinforce this concern. The purpose of this review is to discuss why proper disinfection of equipment is important, why this is challenging in low-resource settings, and suggestions for solutions to move forward.

## 1. Introduction

Despite advances in knowledge and technology, global neonatal mortality rates remain unacceptably high. In the year 2000, the United Nations Millennium Development Goals sought to address the issue of childhood mortality. They set a goal to reduce the number of deaths of children less than five years old by two thirds [1]. By the year 2015, overall childhood deaths were reduced by more than one half; however, the progress in reducing deaths in children aged 1–59 months outperformed the progress in reducing deaths of newborns [2]. As such, neonatal mortality comprises a rising proportion of childhood mortality worldwide [2,3].

The three major causes of neonatal mortality are prematurity, perinatal events, and infection [4,5]. Stakeholders from around the world came together in 2010 to create an educational intervention, Helping Babies Breathe (HBB), to decrease perinatal event mortality by training birth attendants how to resuscitate neonates [6]. Helping Babies Breathe has been implemented in more than 80 countries around the world with more than 500,000 local providers trained [7]. It has been shown to decrease neonatal mortality and improve neonatal care capacity [8,9].

**Figure 1:**
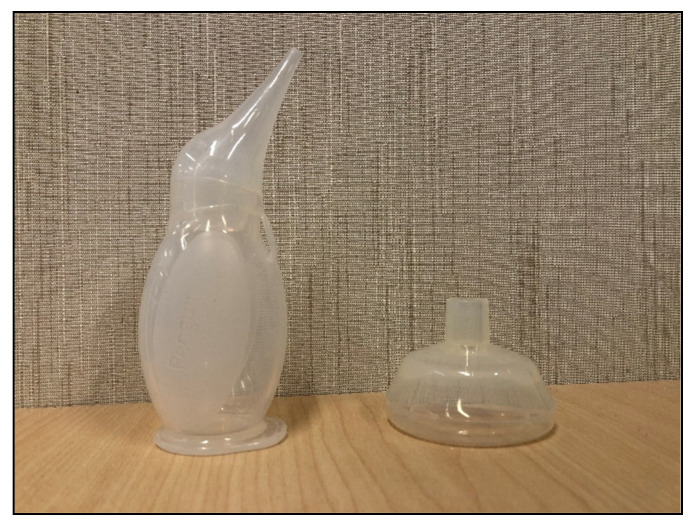
Examples of resuscitation equipment; Image A. White 2020.

With infection as one of the other leading causes, there has been growing interest in ways to mitigate this extensive and complex problem [3,4,5,6,7,10,11]. Clean, functional resuscitation equipment is important to prevent consequences of both perinatal events and infection, and in low-resource settings, there is concern that vital, reused resuscitation equipment undergoes suboptimal disinfection in the newborn nursery (Figure 1). In 2016, PATH, a nongovernmental organization of global health innovators, convened an international group of stakeholders, including members of the HBB editorial group, to write the PATH guidelines for the reprocessing of neonatal resuscitation equipment in resource-constrained settings. These guidelines have not been studied for sustainability and feasibility.

As such, this review covers a brief history of disinfection as well as a summary of the importance of adequate reprocessing and current standards in high resource and resource-constrained settings. Further, we discuss the reality of implementation of the PATH reprocessing guidelines in low-resource settings and some considerations for how to devise a more feasible and sustainable solution as we move into the future for neonates and beyond.

## 2. A Brief History of Disinfection

Standards for disinfection were established by Dr. Earl Spaulding in the 1960s [12]. The Spaulding Criteria describe “a strategy for sterilization or disinfection of inanimate objects and surfaces based on the degree of risk involved in their use” and assign reusable medical equipment to one of three categories: (1) critical; (2) semicritical; and (3) noncritical (Table 1) [12,13]. Critical items are defined as instruments that enter sterile tissues, such as surgical tools; these items require sterilization. Semicritical items come in contact with tissues that are not sterile but are at high risk for transmission of infection, including nonintact skin or mucous membranes. Examples of these type of items include respiratory equipment and anesthesia equipment, and at minimum these items must undergo high-level disinfection (HLD), although sterilization is preferred. According to the Centers for Disease Control and Prevention (CDC), HLD is “a disinfection process that inactivates vegetative bacteria, mycobacteria, fungi, and viruses but not necessarily high numbers of bacterial spores”, while sterilization is “the use of a physical or chemical procedure to destroy all microorganisms including large numbers of resistant bacterial spores” [14]. Neonatal resuscitation equipment falls within the semicritical category, and as such, the current gold-standard is HLD at minimum, with sterilization preferred when possible. Noncritical items, such as stethoscopes and blood pressure cuffs, come in contact only with intact skin or may not directly touch a patient at all; at minimum, these items must undergo low-level disinfection.

## 3. The Importance of Equipment Reprocessing: From Spaulding to PATH

### 3.1. Disinfection of Equipment in High-Resource Settings

**Figure 2:**
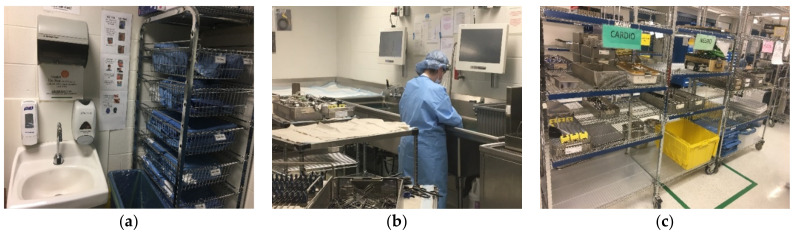
Examples of reprocessing in a high-level medical center: (**a**) Intake area for dirty equipment; (**b**) precleaning of dirty equipment; and (**c**) reprocessed equipment in storage. Images A. White, 2018.

Medical equipment is manufactured as either single- or multi-patient use. Most hospitals employ a combination of both. Disinfection methods often follow manufacturer recommendations, which are based on Spaulding criteria and CDC guidelines. In high-resource places, including many settings in the United States, single-use equipment is disposed of following a single use. This is especially true of neonatal resuscitation equipment. For example, a pediatric team is called for the delivery of a baby for nonreassuring fetal heart tones. The baby is apneic at delivery and receives positive-pressure ventilation using a bag-mask device. Following resuscitation of the newborn, the mask is thrown away. Much of the reusable equipment used in healthcare settings, including surgical instruments, undergoes sterilization. Certain equipment, including endoscopy tools, may undergo HLD rather than sterilization. There are multiple steps involved in reprocessing, including: (1) precleaning; (2) cleaning; (3) inspection and test of function; and (4) storage. Many substeps also take place (Table 2). Reprocessing in high-resource settings is conducted in an area with dedicated space, equipment, and trained personnel (Figure 2). Supplies including cleaning detergents and brushes are consumed quickly. Elements such as clean running water and continuous electricity are essential to the process and commonly taken for granted.

### 3.2. Disinfection of Equipment in Low-Resource Settings

As previously mentioned, there is a lack of evidence regarding recommendations for reprocessing in low-resource settings specifically. As such, a team of experts gathered and published a multistep consensus algorithm for reprocessing of reusable neonatal resuscitation equipment; no standard recommendations exist for reprocessing single-use equipment [15,16]. The guideline follows the gold-standard for reprocessing of semicritical equipment as set by the CDC and goes through four main stages to achieve sterilization or HLD: (1) preparation; (2) predisinfection; (3) disinfection; (4) and postdisinfection (Table 3) [15]. Three options are presented for disinfection: (1) chemical HLD with chlorine or activated glutaraldehyde; (2) heat HLD by boiling or steaming; and (3) sterilization by autoclave [15]. This issue was felt to be of such importance that the guideline was published with the second edition of HBB, with reprocessing teaching recommended in addition to the main resuscitation curriculum [6]. However, it was quickly noticed that the algorithm called for several assets often lacking in low-resource areas, including materials, personnel, time, and physical space, not to mention a supply of clean, running water as well as a reliable source of electricity if choosing heat HLD or sterilization.

Neonatal lives could be saved if more sustainable, equitable access to lifesaving equipment was made available wherever babies are born. This includes proper disinfection resources. Historic case reports from neonatal care settings raise concerns that the lack of effective reprocessing increases infection rates in this vulnerable population [17,18]. In *Resuscitation* in 2015, Eslami et al. wrote a letter to the editor regarding the reprocessing of neonatal resuscitation equipment based on observations they made while implementing HBB in Kenya [6,19]. They noted that equipment was being reprocessed improperly leading to staining and leftover residual sticky material. In some cases, the equipment was rendered altogether ineffective for resuscitation of a newborn [19]. The authors made the following call to action:

“In the short-term, implementers of [Helping Babies Breathe] and other [neonatal resuscitation] courses should ensure that the following concepts and skills are integrated into training and quality improvement efforts: (a) emphasis on the importance of proper reprocessing of all reusable [neonatal resuscitation] commodities, regardless of device manufacturer or what cleaning/disinfection methods are used; (b) active practice to disassemble and reassemble the resuscitator…; (c) proactive identification and discussion of local, context-specific gaps, barriers, and solutions to reprocessing. In the longer term, our findings suggest that issues surrounding reprocessing of [neonatal resuscitation] commodities should be emphasized in the upcoming revision of the [Helping Babies Breathe] curriculum [19].”

This editorial brought the issue of inadequate reprocessing of neonatal resuscitation equipment, and the consequences thereof, to international attention. Discussions garnered the attention of PATH as well as leaders within the HBB community. Multiple stakeholders, including the United States Agency for International Development, were convened to develop reprocessing guidelines.

In literature review at that time, there was a lack of evidence regarding recommendations for reprocessing of resuscitation equipment in low-resource settings specifically. As such, a team of experts was gathered, and recommendations were developed based on the best available evidence, including CDC and World Health Organization guidelines, as well as consensus opinion. The result was a multistep algorithm to attain sterilization or HLD, with specific design for use in resource-limited settings [15].

The recommendations were published by PATH and then incorporated into the 2nd Edition HBB curriculum, to be included in trainings of birth attendants around the world [6]. However, there is concern that even this revised algorithm may be too daunting for the locales for which it was designed, with inadequate resources to comply.

While the PATH guideline is the first reprocessing protocol for neonatal resuscitation equipment designed for use in low-resource settings, it is unclear if it is feasible or sustainable in low-resource settings. This is largely due to the many barriers to adequate disinfection in resource-strained areas, including: (1) reuse of single-use devices; (2) newborn nurseries that are overcrowded and understaffed; (3) lack of clean and/or running water; and (4) substandard disinfection practices including hand hygiene [20].

## 4. Reprocessing Realities in Kenya and Malawi

**Figure 3:**
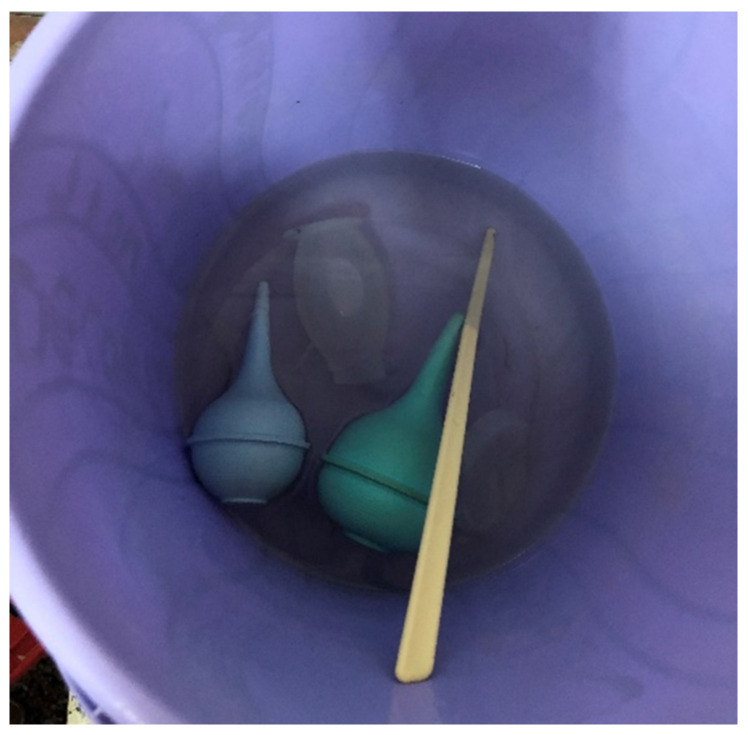
Combination of multi- and single- use equipment undergoing chemical disinfection in a maternity ward in Kenya. Image A. White, 2020.

Medical equipment in resource-limited settings is commonly a combination of single- and multi-use. This is due to supply chain challenges, charity/humanitarian practices, and equipment cost [21,22,23,24]. There is questionable need for different reprocessing tactics between the different equipment types, as it is unclear if single-use equipment would withstand the rigorous disinfection suggested in the PATH guidelines. As a result, current disinfection practices in many resource-constrained settings employ chemicals, either chlorine or activated glutaraldehyde (Figure 3), over heat or other options.

In addition to understanding the best method of disinfection to use, systematic changes are needed to support personnel dedicated to the task of reprocessing and to help set them up for success. In high-resource settings, there are designated personnel and space for reprocessing. In many resource-constrained settings, the same personnel responsible for caring for mothers and babies are also responsible for reprocessing. Likewise, there may be a lack of designated space for reprocessing. Even if there is a designated space, it may be in the same area deliveries are happening, which may make the use of a heat source a safety concern.

**Figure 4:**
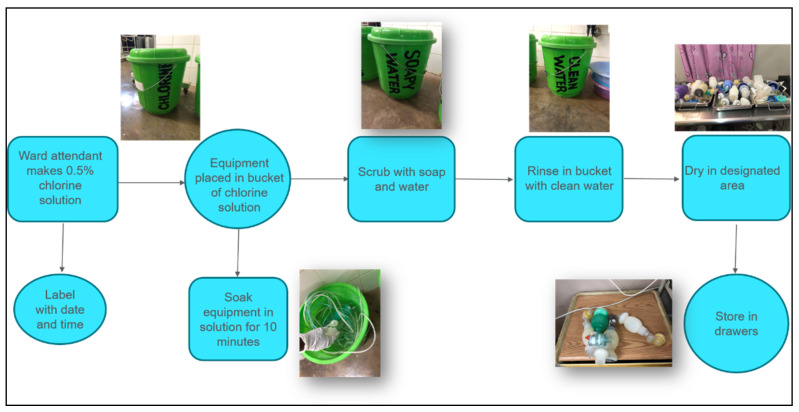
Process map of reprocessing of neonatal equipment at a hospital in Malawi.

Observations by our neonatal team at a busy tertiary referral center in Malawi and an active rural referral hospital in Kenya found that disinfection of nursery equipment is the responsibility of either nonmedical personnel, such as ward attendants, or medical personnel, including nurse midwives, all of whom are committed to a number of additional tasks in addition to reprocessing. Hospital policies in both places (Malawi and Kenya) dictate a chemical method of disinfection such as the following process in place in the nursery in Malawi: (1) submerge used equipment in a chlorine solution for 10 min; (2) scrub equipment in a bucket of soapy water; (3) rinse in bucket of clean water; 4) dry; and (5) store (Figure 4).

**Figure 5:**
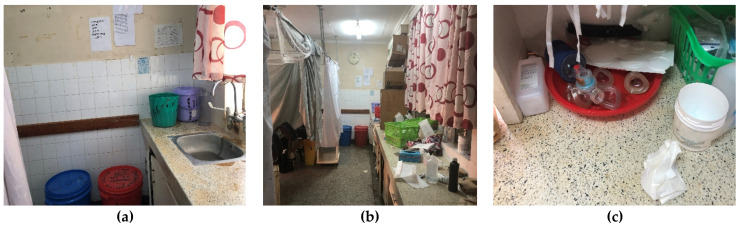
Reprocessing of neonatal equipment in a hospital in rural Kenya. (**a**) Reprocessing area with buckets for different steps; (**b**) reprocessing area at the end of hallway, with active labor and delivery beds in the image to the left; and (**c**) reprocessed neonatal resuscitation equipment in the red tray, awaiting patient use. Images A. White, 2019.

At the hospital in rural Kenya, reprocessing of equipment in the labor and delivery ward is also performed with chemicals. The reprocessing space is small and cramped and primarily serves as a patient-care area (Figure 5). Clean, filtered water is rationed by department, with a small allotment dispensed to meet all department needs with a monetary cost associated with each volume.

**Figure 6:**
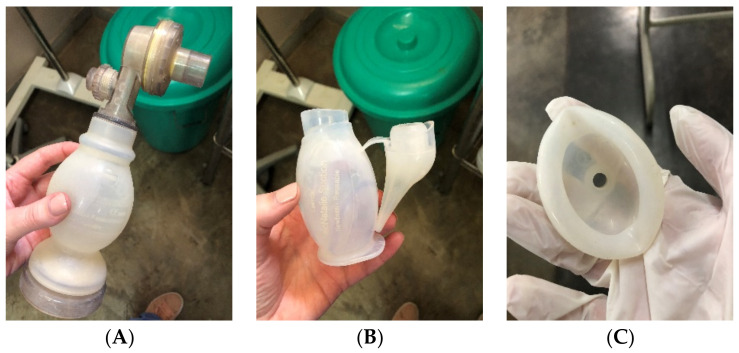
Reprocessed equipment in a referral hospital in Malawi. (**A**) Disinfected bag-mask device, (**B**) disinfected suction bulb, and (**C**) disinfected, warped mask. Images A. White, 2018.

Reprocessing is conducted by nurse-midwives also responsible for managing inpatients, active deliveries, and neonatal resuscitations. Used equipment may be left in the chemical solution beyond the recommended submersion time. while staff are busy with other tasks. Alternatively, the equipment may be removed from cleaning solution prematurely due to its urgent need for use to resuscitate a sick newborn; without being properly rinsed and dried, the neonate is exposed to chemicals and potentially to remaining infectious material. In both Kenya and Malawi, it was commonly reported by the local staff that there were challenges with complying with the minimal disinfection guidelines already in place: there were no personnel dedicated to the task of reprocessing; cleaning solutions were not being changed at the recommended frequency; equipment was being left in the solutions for longer than recommended or being removed before the recommended time; the equipment was not being disassembled prior to reprocessing; and there was no clean and practical place to store the disinfected equipment (Figure 6). At both sites, there was interest in improving this situation with a simple, effective reprocessing method that would also promote feasibility and sustainability.

Prior to a PATH guideline implementation trial, we sought to gather baseline opinions about reprocessing and any perceived barriers from our Kenyan partners, including nurse midwives, an obstetric nurse matron, a nursery nurse matron, the local pediatric consultant (attending), and the hospital’s sole respiratory therapist. They identified numerous concerns. First, they were concerned about the cumbersome process of disassembly of the bag-mask device (it disassembles into 10 pieces), the risk of losing the small pieces once disassembled, and the potential for high rate of error in reassembling the bag at the conclusion of the process. The rural Kenyan hospital was so concerned about this that they only agreed to an implementation trial of the new guideline if the bag-mask device could stay intact.

The second major concern of our Kenyan colleagues was the constant scarcity of the preferred, reusable equipment. They reported the challenges of gaining access to affordable equipment supply chains and reliance on single-use equipment to fill in the gaps. There are many barriers to establishment of a successful supply chain [21,22,23,24], and as such, hospitals frequently rely on donated equipment [21]. This equipment is often a mix of single- and multi-use, and in some cases, has passed its expiration date. Reliance on donations leads to irregular deliveries of supplies and dependence on unequitable partnerships; also, it is often not sustainable [24]. Supply chain issues and reliance on donations furthers the challenges of infection control of single-use equipment, an issue yet to be sufficiently investigated or mitigated.

Recent work by Gilbertson et al. demonstrated adequate disinfection of reusable equipment using heat methods; however, heat methods may be destructive toward single-use equipment while outstanding questions remain about the effectiveness of chemical HLD [25]. Furthermore, when equipment was submerged in chlorine-based solutions for a long period of time, some of the equipment was rendered inoperable; this has been observed by us in the field, including at the Malawian and Kenyan sites (Figure 6C). This is particularly concerning as many sites in Africa employ chlorine for disinfecting equipment and have few resources to replenish nonfunctional equipment. Thus, the final concern raised by Kenyan colleagues was whether their equipment could survive more intense reprocessing. After this discussion, the central question was clear: what can be done to simplify reprocessing of vital equipment to promote feasibility and sustainability, while not sacrificing effectiveness in resource-constrained settings?

## 5. Considerations for the Future of Reprocessing in Resource-Constrained Settings

To move forward, infection control advocates should consider three interrelated action steps: (1) engagement and partnership with front-line providers to help revise guidelines for their settings, (2) simplifying the PATH guidelines to improve feasibility, and (3) maintaining efficacy and sustainability through quality control.

### 5.1. Engagement of Front-Line Providers for Front-Line Solutions

It is likely that there is no one-size-fits-all answer to the question of which reprocessing method is “best”. The method needs to be appropriate to local settings, driven by local providers, and tailored to local resources and local needs. For example, as heat HLD by steaming has been shown to be effective [25], steaming may be a popular and practical method of disinfection to use in many places in Asia, where steaming rice and vegetables is a culturally common practice. Steaming is not common in many places in Africa. Our team was unsuccessful in finding appropriate steaming equipment, including a pot and steaming pan, in a small town in rural Kenya. At our site in Kenya, boiling is more familiar and practical than steaming, and equipment for boiling is readily found. Taking a break for chai tea is a cultural staple in many Kenyan communities, and as such, hot plates, to boil water for tea or to boil water for disinfecting equipment, are easily found in stores. For even more resource-limited places, including those without a consistent source of electricity, a more simplified method of cleaning may be preferred and needs to be identified through further study.

The experts on these barriers are the nurse-midwives, physicians, and ward attendants who are performing reprocessing and caring for women and newborns in these settings every day. Infection control advocates from high-income settings should engage and partner with front-line providers to discuss barriers, work-arounds, and creative innovation in context. Likewise, partnership and inclusion of front-line stakeholders from low-resource settings also can push conversations about ethical equipment donation and begin to address the challenge of single-use equipment reuse and supply chains [26]. Finally, a lack of dedicated staff for reprocessing is a key barrier, often due to the result of a lack of funding. In the future, infection control partners from high-income settings could consider global health organizational and academic institutional partnerships that could contribute to capacity building in infection control.

### 5.2. Simplification of the PATH Guidelines for Feasibility

To simplify the PATH algorithm, a few ideas may be considered to start. First, targeting the most contaminated parts of the equipment may increase feasibility. If we assume that the mask is the most contaminated part, since it interfaces directly with the newborn, could we disconnect and reprocess only the mask—would that be enough to reduce transmission of infection? This seems insufficient, as the equipment is not truly undergoing adequate reprocessing. Second, structural simplification of the bag-mask device could promote ease of disassembly and proper disinfection. Perhaps engineering a simpler bag-mask device with fewer pieces and/or invention of a bottle-like brush or other device might allow for cleaning without disassembly.

As other options for simplifying, certain step(s) of the algorithm could be altered or eliminated. Finally, whether recommendations can be made to safely reprocess devices intended for single-patient use is an outstanding issue. With the high prevalence of single-use equipment in many resource-limited areas, single-use equipment must be considered in reprocessing methods.

In addressing some of these ideas, laboratory simulation can be helpful. Simulation may be used both to develop specific methodology needed for testing, as well as to test interventions [27]. A clean, controlled setting can facilitate the testing of hypotheses under ideal circumstances, with thoughtfully developed models that can then be translated to the field. At the same time, it is important to balance this with field trials and the vital perspectives of front-line workers in low-resource facilities who best understand the feasibility and sustainability of these guidelines. Recommendations about reprocessing are not only derived from science but around cultural and institutional preferences that influence behavior change. With input from stakeholders both domestically and abroad, there is need for the development of reprocessing procedures that are simple, effective, and sustainable.

### 5.3. Quality Assurance of Reprocessing in Resource-Constrained Settings

Quality assurance is a critical consideration for any reprocessing program and especially in resource-constrained settings [28]. Any modification of the PATH algorithm, or alternative disinfection process, must be comparable in efficacy to the gold-standard method in order to be acceptable and recommended for use. To test efficacy, a reliable and reproducible method must be used. This proves to be difficult considering the design of the equipment; with irregular surfaces and tight crevices, it is challenging to sufficiently test. A few methods to determine bacterial burden from equipment have been tried. Adenosine triphosphate (ATP) counts are a quantitative method of measurement that can be performed by a handheld, battery-operated machine to report the presence of all live organisms on a surface rather than specific culturable bacteria. The advantages of ATP counts include ease of use, low cost, and quick (< 1 min) and easy to interpret results, precluding the need for a sophisticated microbiology laboratory [29,30]. An alternative method of quantitative measurement to evaluate bacterial burden is to count colony-forming units of bacteria per centimeter squared. This method has been challenging to use for several reasons, including the structural design limitations mentioned above. Although there are challenges, obtaining colony-forming units per centimeter squared from equipment has been successfully achieved, as demonstrated in publications by Zemitis et al. [31] and Frickmann et al. [32]. Regardless of the method used for monitoring of reprocessing, quality assurance check lists, plan for ongoing monitoring, and feedback from front-line providers need to be incorporated into revised reprocessing guidelines.

## 6. Conclusions

The publication of the PATH guideline was a start in addressing a considerable issue: how best to reprocess neonatal resuscitation equipment in low-resource settings. There are outstanding questions about how to modify the guideline into something that is feasible and sustainable while maintaining efficacy. The ideal process needs to be practical with regards to space, time, and staffing barriers faced by many low-resource facilities with input from front-line stakeholders. Local partnership and leadership are essential. Abandonment of a one-size-fits-all model facilitates success, with recognition that preferred and feasible methods of disinfection vary from site to site. In addition, application of a process to address disinfection of the myriad other types of hospital equipment is needed, and this may be a starting point. Further efforts need to be made to make implementation cost effective and easy to learn so that staff of a variety of education levels can participate and sustain change. System-wide change is needed, with leadership encouraging and supporting change. More evidence is needed from the field regarding possibilities and barriers. This work is urgently needed, and as this generation has already risen to meet the challenge of the COVID-19 pandemic, it also has the potential to make a significant impact in reducing neonatal infections and reducing neonatal deaths now and for years to come.

## Figures and Tables

**Table 1:** The Spaulding criteria [13].

Classification	Definition	Level of Reprocessing	Examples
Critical	Enters sterile tissue	Sterilization	Surgical instruments
Semi-critical	In contact with nonintact skin or mucous membranes but does not penetrate them	Minimum high-level disinfection Sterilization preferred	Respiratory equipment Anesthesia equipment
Noncritical	Touches only intact skin, or does not directly touch patient	Low-level disinfection	Electrocardiogram machine Oximeter Bedpan

**Table 2:** Example of sterile reprocessing at a high-level hospital in the United States.

Stage	Steps
Precleaning	1. Rinse with water 2. Clean with enzymatic solution 3. Rinse with distilled water
Cleaning	1. Ultrasonic cleaner 2. Automatic washer
Inspection and Test of Function	1. Assemble equipment 2. Package 3. Test function 4. Place indicator for sterilization
Sterilization	Steam preferred
Storage	Store in clean, dry place

**Table 3:** Reprocessing steps according to the PATH guideline [15].

Stage	Steps
Preparation	1. Wear complete personal protective equipment 2. Clean reprocessing area 3. Prepare reprocessing materials 4. Label containers
Predisinfection	1. Preclean 2. Disassemble 3. Clean 4. Rinse (Remove limescale if needed) 5. Dry before sterilization or chemical disinfection
Disinfection	1. Disinfect by: a. Chemical HLD b. Heat HLD c. Sterilization 2. Dry
Postdisinfection	1. Inspect 2. Reassemble 3. Test function 4. Store

## Data Availability

Not applicable.
